# Traditional online and live-streaming dual-channel strategies and pricing policies

**DOI:** 10.1371/journal.pone.0311385

**Published:** 2025-01-08

**Authors:** Yue Yu, Songshi Shao, Mingli Yuan, Minghe Sun

**Affiliations:** 1 College of Transportation Engineering, Dalian Maritime University, Dalian, China; 2 College of Naval Architecture and Ocean Engineering, Naval University of Engineering, Wuhan, Chian; 3 School of Business Administration, Northeastern University, Shenyang, China; 4 Department of Management Science and Statistics, The University of Texas at San Antonio, San Antonio, Texas, United States of America; Shandong University, CHINA

## Abstract

This study examines the pricing policies and the dual-channel strategies in a two-tier supply chain where a manufacturer distributes products through a retailer and considers the opening of a traditional direct online channel or a live-streaming sales channel The manufacturer has three sales modes, i.e., traditional retail channel only, opening a traditional direct online channel and opening a live-streaming direct sales channel, to consider. The manufacturer and the retailer play a Stackelberg game wherein the manufacturer has the first mover advantage. The two supply chain members can follow two decision sequences where the manufacturer decides the direct online retail price before the retailer does in one, and the manufacturer and the retailer cooperate to decide the retail prices simultaneously in the other. The profit optimization models for the three sales modes are formulated, and the closed forms of the optimal solutions are then derived through backward induction. The optimal pricing decisions and profits with different decision sequences are compared and analyzed among the three sales modes. The results suggest that the manufacturer should introduce a live-streaming direct sales channel when facing fierce competition, and should not introduce a dual channel to create a win-win situation for the two supply chain members when facing mild competition. The manufacturer should increase the wholesale and the direct online retail prices and make more live-streaming sales effort, and the retailer should also increase the retail price, if the streamer has good sales skills when introducing a live-streaming sales channel. The manufacturer and the retailer should decide the retail prices simultaneously when a live-streaming sale channel is introduced.

## 1. Introduction

Over the last several years, live-streaming e-commerce as a rapidly emerging sales mode has experienced explosive growth and turned into a new driver of economic development. Research reports showed that live-streaming sales in the United States, with $50 billion in 2023, were projected to grow by 36% by 2026 (https://www.statista.com/statistics/1276120/livestream-e-commerce-sales-united-states/). A Statistical Report on China’s Internet Development showed that the number of live-streaming e-commerce users in China reached 526 million as of June 2023, an increase of 11.94 million from December 2022, accounting for 48.8% of the total Internet users (https://www.cnnic.cn/n4/2023/0828/c88-10829.html). The rapid expansion of live-streaming e-commerce users drives a substantial increase in the transaction volume of the e-commerce market. According to data, the total size of live-streaming e-commerce in China is likely to reach $335 billion by 2025 from $190.57 billion in 2021 (https://www.iimedia.cn/c400/86233.html). This rapid explosive growth means that the live-streaming e-commerce market has a huge potential and opportunity for development, and the market size is expected to continue to expand in the coming years. Thus, the live-streaming sales mode has generated widespread interest across businesses and academia.

In reality, numerous manufacturers not only supply products to retailers but also sell products directly to consumers. Some manufacturers are actively collaborating with prominent online celebrities or social influencers to sell branded products through live-streaming platforms. For instance, L’Oréal Paris collaborated with the beauty influencer Kristen Leanne for a live-streaming event on her Instagram account to showcase and promote makeup products (https://fieldmarketing.com/news/urban-decay-appoints-flash-pack-kristen-leanne-launch-party/), and Estée Lauder hired the well-known streamer Li Jiaqi in China to sell its products via live streaming on Taobao (https://post.55haitao.com/show/205191/). Compared to the traditional online sales mode, the live-streaming sales mode shows product characteristics and effects more intuitively and vividly through streamer explanations of products and trial demonstrations. It also offers a real-time interaction where consumers can instantly interact, ask questions and make comments with the streamer, making a more lively and personalized shopping process [1]. As a result, live-streaming sales can reduce consumer perceived uncertainty about products, delivering them with an extra level of value [2].

However, manufacturers adopting a live-streaming sales mode may experience certain challenges. On the one hand, a manufacturer introducing this sales mode may lead to intense competition with the retailers in its distribution channels, resulting in reduced orders from the retailers, and subsequently affecting its performance. In practice, MAC and Clinique frequently conduct live-streaming sales on social media platforms such as Tiktok and Taobao (https://business.sohu.com/a/600465721_121123784), potentially affecting the orders from the retailers in the market. On the other hand, employing streamers and using live-streaming platforms incur, but traditional online operations do not typically generate, specific additional costs. These additional costs may negatively affect the manufacturer’s profit. For instance, a manufacturer spent ¥0.8 million hiring celebrity Li Xiang to sell mink coats through live streaming, resulting in zero revenue (https://www.163.com/dy/article/ESLA7K0G0514R9OJ.html), and a certain official media spent ¥3 million on a live-streaming platform, but bringing in a total sales of only ¥1.385 million (https://m.jiemian.com/article/7239527.html), demonstrating a complete lack of cost-effectiveness. Moreover, this sales mode incurs some inconvenience costs for consumers, such as waiting and watching the live streaming. The live streaming for specific products typically occurs at specific times of the day, causing some time sensitive consumers reluctant to purchase via live streaming. It is currently unclear whether manufacturers can truly generate profits by introducing live-streaming sales. The impact of the introduction of live-streaming sales on the retailer’s pricing policy also needs investigation since the downstream retailers are affected.

The above observations motivate the investigation of the impacts of the introduction of a dual channel on the supply chain performance. In this work, three different, i.e., not setting a dual channel (NS), setting a traditional online direct sales channel (SO), and setting a live-streaming direct sales channel (SL), strategies or sales modes are considered for the manufacturer. The manufacturer and the retailer are assumed to have two different decision sequences when developing a dual channel. One is when the manufacturer decides the direct online retail price before the retailer does, and the other is when the manufacturer works with the retailer to decide the retail prices simultaneously. The following four research problems are investigated: (1) What are the optimal pricing decisions for the manufacturer and the retailer under the three strategies? (2) Should the manufacturer introduce a live-streaming direct sales channel? (3) Which strategy benefits the retailer? (4) Which decision sequence is appropriate for the manufacturer and the retailer?

To resolve the above four research problems, this study considers a two-tier supply chain consisting of a manufacturer and a retailer. The manufacturer supplies products to the retailer, who then sells them to consumers, and considers the opening of a dual channel. For the three, i.e., NS, SO and SL, strategies, the two supply chain members play a Stackelberg game in which the manufacturer has the first mover advantage when they follow the two decision sequences. The profit optimization models are developed, and the closed forms of the optimal solutions are then derived using backward induction. Sensitivity analyses of the optimal decisions are performed when the values of the key parameters change. The optimal decisions and profits are compared for the three strategies or sales modes with the different decision sequences to determine each member’s best sales mode.

This study makes the following three contributions to the related literature. First, the conditions for the manufacturer to introduce live-streaming sales are examined for the first time as it develops a direct sales channel to compete with the retailer. The related literature examined the live-streaming strategy from the perspective of the retailer or e-commerce platform, but rarely from the perspective of the manufacturer [1,3]. Second, three different manufacturer dual channel strategies are examined. Previous works investigating live-streaming sales usually investigated two strategies, i.e., introducing or not introducing live-streaming sales, ignoring the investigation of whether other sales modes are the best option when live-streaming sales is not introduced [4,5]. Finally, two decision sequences are considered for the live-streaming strategy in a dual-channel supply chain for the first time.

The rest of this work is organized as follows. The relevant literature is reviewed and the contributions are highlighted in Section 2. The problem is described and the models are formulated in Section 3. The optimal results with the different decision sequences for the three strategies are analyzed in Section 4. The optimal decisions and profits in the three strategies are compared in Section 5. Several numerical experiments are conducted in Section 6. The main research results are summarized and future research directions are stated in Section 7. All proofs of propositions and corollaries are provided in the S1 Appendix.

## 2. Literature review

This work is related to three streams, i.e., dual-channel supply chains, channel selection, and live-streaming sales. These three streams of literature are reviewed separately in this section.

Dual-channel supply chains have been a significant topic of research over the past few decades [6,7]. The studies related to dual-channel supply chains focused on pricing policies, decision sequences and consumer behaviors, among others. Several studies investigated pricing policies for dual-channel supply chains and examined the effects of different factors on pricing decisions and outcomes. Hamzaoui et al. explored the effect of consumer loyalty to the retail channel on pricing decisions, and showed that consumer loyalty significantly affects market shares and pricing policies[8]. He et al. developed four two-period pricing models under different market conditions, and studied the impacts of the different market conditions on pricing policies for a dual-channel supply chain [9]. Qiu et al. studied pricing decisions for a dual-channel supply chain with showrooms and showed that showrooming could help brick-and-mortar retailers mitigate profit losses [10]. The supply chain member decision sequences also significantly impact the performance of a dual-channel supply chain. Chun and Park developed Stackelberg game models with different decision sequences, and investigated the appropriate decision sequence for the supply chain members [11]. Israeli et al. further examined the sequences of dynamic pricing decisions for an automotive supply chain [12]. Tseng et al. investigated decision sequences in a bi-level market, and suggested that the wholesaler should lead in the decision-making process while the manufacturer should propose a profit-sharing contract in a deregulated system [13].

The channel selection strategy is also a crucial area in supply chain management. Some works focused on channel selections between online and traditional channels, and showed that online channels generate more profits for suppliers than traditional retail channels. Gilbert et al. found that channel competition influenced substitution rates between traditional and online channels, and emphasized the dynamic interaction between different sales channels [14]. Wang et al. highlighted that the supplier’s decisions in online channels depended on the proportion of unit operational costs and income distribution across different channels. This result suggests that profit considerations play a critical role in channel selections [15]. Li et al. considered the disadvantages of direct selling and further investigated channel sharing strategies, and their work contributed to the understanding of how suppliers balance between direct and shared channels [16]. Zhang et al. examined channel selection with information sharing, and showed the importance of information flow in channel selection strategies [4]. Ma et al. developed two game models, divided consumers into two, i.e., uniqueness and uniformity, types, and investigated the influences of these consumer types on channel selection strategies [17]. Additionally, Goraya et al. integrated online and offline channels, and investigated the effects of consumer channel preferences on channel selection decisions among offline, online and integrated channels [18].

Selling through live streaming has attracted extensive attention from both academics and practitioners. Various studies examined the influence of streamers on consumer behavior through empirical research. Wang et al. collected a rich live-streaming dataset to examine the impacts of two categories of, i.e., social interactions-transaction-oriented and relationship-oriented, streamers on consumer behavior [19]. He et al. explored the key factors influencing consumer live-streaming shopping experiences to identify critical areas for enhancing user satisfaction [20]. Moreover, some works investigated the live-streaming sales strategies using game models. For example, Hua et al. developed game models to examine the impacts of live-streaming sales on consumer purchasing decisions [21]. Additionally, some works examined the impacts of live-streaming e-commerce on the business operations strategies using game models. Zhang, Chen, et al. investigated optimal live-streaming strategies for a single firm streamer framework. Notably, Zhang, Chen, et al. assessed the feasibility and implications of introducing live-streaming sales for a supply chain, and focused on live-streaming sales with an agency sales mode. Furthermore, some works focused on operations strategies with live-streaming sales [22]. Jin et al. examined optimal cooperation strategies with streamers [23], and Zhen et al. focused on pricing and quality decisions [24]. Da et al. investigated sales modes and pricing strategies [25].

Although a host of existing works examined various aspects of dual-channel supply chains, selling through live streaming has received relatively little attention. Table 1 summarizes the contributions of the relevant studies and shows the differences between this study and the previous works. The existing literature predominantly focused on pricing decisions, decision sequences and channel selection strategies, with limited exploration of the integration of live-streaming sales into dual-channel supply chains. Hamzaoui et al.investigated the pricing decisions in a dual-channel supply chain [8]. Fukuyama et al. further considered the different decision sequences in a dual-channel supply chain [26]. Zhang et al. examined channel selection between the dual channel and other channel structures [4]. The above studies did not investigate the introduction of a live-streaming sales channel. Zhang et al., Chen et al. and Huang et al. investigated the live-streaming channel from the perspective of the retailer and e-commerce platform rather than the manufacturer in a supply chain [1,3,22]. Du et al. investigated the live-streaming strategy from the perspective of the manufacturer, but did not examine the price competition between the retailer and the manufacturer and neglected the wholesale price decision [27]. This study extends the above works in three ways. (1) The conditions for the manufacturer to introduce a live-streaming direct sales channel are investigated in a dual-channel supply chain. The competition between the direct sales channel and the retailer retail channel is considered. (2) The three, i.e., NS, SO and SL, strategies are considered for the manufacturer. (3) The two supply chain members are assumed to follow two decision sequences, where one is when the manufacturer decides the direct online retail price before the retailer does, and the other is when the manufacturer collaborates with the retailer to decide the retail prices simultaneously.

**Table 1:** Comparison of the related works.

Literature	Dual-channel	Decision sequence	Channel selection	Live-stream selling	Key contributions
Fukuyama et al. (2023) [26]	✓	✓			Estimated the degree of firm input market power via data envelopment analysis.
Qiu et al. (2024) [10]	✓				Studied the impacts of consumer showrooming behavior in a dual-channel supply chain.
Hamzaoui et al. (2024) [8]	✓				Explored the effect of consumer loyalty to the retail channel on pricing decisions.
Zhang et al. (2023) [4]	✓		✓		Investigated the influences of e-commerce on the behaviors of suppliers and brick-and-mortar retailers.
Du et al. (2023) [27]				✓	Developed the manufacturer’s live-streaming strategy.
Ma et al. (2023) [17]	✓		✓		Investigated the co-branding strategies in a platform-based supply chain.
Zhang et al. (2024) [28]				✓	Studied the live-streaming strategy for an e-commerce platform supply chain.
Chen et al. (2023) [1]				✓	Studied the conditions for an online retailer to introduce a live-streaming channel.
Huang et al. (2024) [3]				✓	Explored the live-streaming strategy for two competitive retailers.
This study	✓	✓	✓	✓	Investigates the live-streaming strategy for a manufacturer in a dual-channel supply chain with different decision sequences.

## 3. Problem description and model formulation

**Fig 1:**
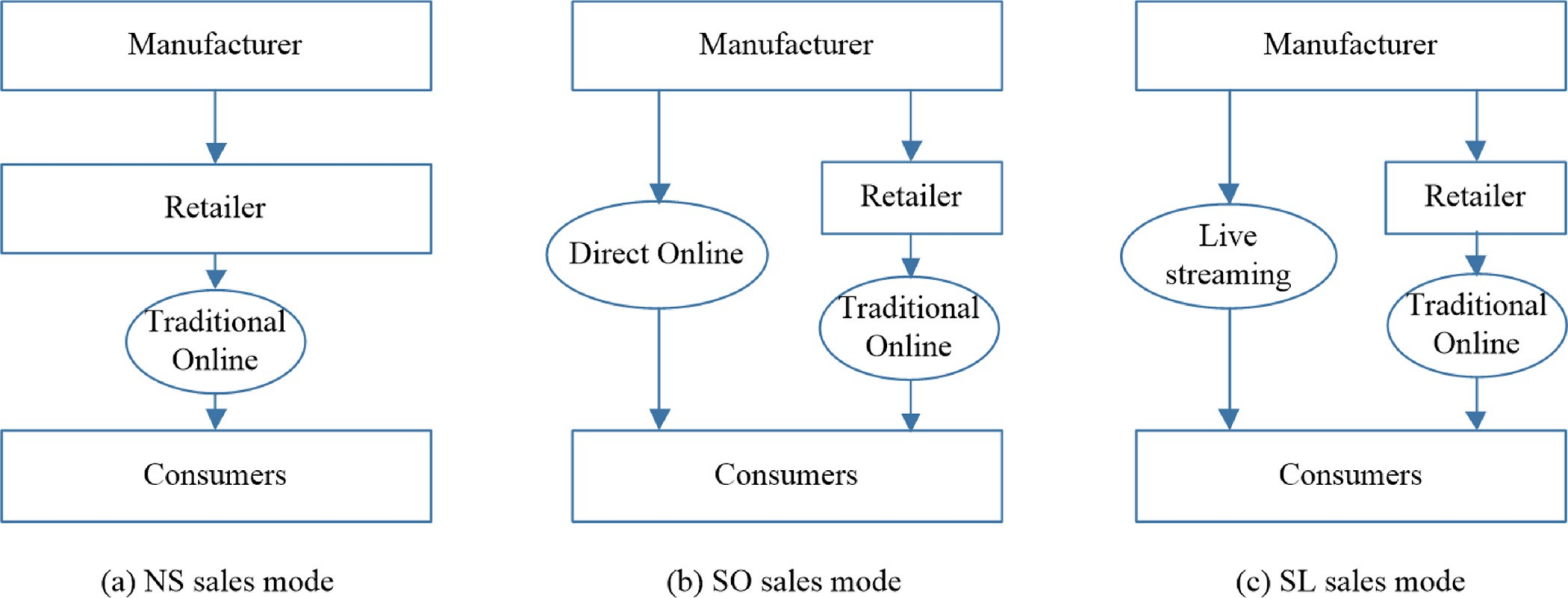
Supply chain structures for the three strategies.

A two-tier supply chain composed of a manufacturer and a retailer is considered. The manufacturer distributes products through the retailer, who sells them to consumers through a traditional online channel. The manufacturer has three, i.e., NS, SO and SL, strategies or sales modes to consider. The supply chain structures for the above three strategies are depicted in Fig 1. For easy reference, the superscripts *NS*, *SO* and *SL* are used to distinguish the three strategies, the subscripts *m* and *r* are used to represent the manufacturer and the retailer, and the subscripts *a* and *b* are used to indicate the two sequences of events in the notations. The notations used in this study are summarized in Table 2.

**Table 2:** Notations and descriptions.

Parameters	Descriptions
*a*	Market potential, *a*>0
*θ*	Retailer’s market share, 0≤*θ*≤1
*β*	Competition intensity, 0<*β*<1
*γ*	Streamer’s sales skills
*κ*	Sales effort level cost coefficient
$D_{j}^{i}$	Channel demand of supply chain member *j* in strategy *i*
$\pi_{j}^{i}$	Profit of supply chain member *j* in strategy *i*
Decision variables	
$p_{j}^{i}$	Retail price of supply chain member *j* in strategy *i*
*w* ^*i*^	Wholesale price in strategy *i*
*e*	Live-streaming sales effort level

Notes: *i*∈{*NS*, *SO*, *SL*} and *j*∈{*r*, *m*}.

In the NS strategy, the manufacturer only supplies products to the retailer at a unit wholesale price *w*^*NS*^, and the retailer then sells them to consumers at a retail price *p*^*NS*^ in a traditional online store. The retailer is assumed to be capable of fulfilling market demand without carrying inventory. In practice, this demand certainty means that the retailer places orders with the manufacturer based on actual demands. Then, the manufacturer produces the products accordingly. This order fulfillment strategy usually applies to products manufactured on demand or those with low costs and short delivery times, such as books, software or other digital products [29]. Following Du et al. [27], the retailer’s traditional online channel demand can be expressed as a linear function $D^{NS}=a-p_{r}^{NS}$, where *a* is the market potential.

In the SO strategy, the manufacturer also sets up an online direct sales channel while distributing products through the retailer. The manufacturer sells the products directly to consumers through its direct online store at a unit direct online retail price $p_{m}^{SO}$, and the retailer sells the products to consumers at a unit retail price $p_{r}^{SO}$ in the traditional online store. In addition, the two supply chain members compete on their retail prices. Thus, the demand of each channel is affected simultaneously by the retail prices $p_{m}^{SO}$ and $p_{r}^{SO}$ of the two channels. Moreover, a portion of the market share is taken by the manufacturer since the direct online channel is introduced. Following Xu et al. and Du et al. [27,30], the demand functions for the retailer and the manufacturer can be expressed as

$$D_{r}^{SO}=\theta a-p_{r}^{SO}+\beta p_{m}^{SO},$$ (1)

$$D_{m}^{SO}=(1-\theta )a-p_{m}^{SO}+\beta p_{r}^{SO},$$ (2)

where *θ* is the retailer’s market share with *θ*∈[0,1], and *β* is the competition intensity between the retailer and the manufacturer with *β*∈(0, 1). The market is monopolized by the retailer if *θ* = 1, or by the manufacturer if *θ* = 0. The competition between the retailer and the manufacturer is virtually absent when *β* approaches 0, and is extremely fierce when *β* approaches 1. A higher *θ* means that the retailer has a larger market share and the manufacturer has a smaller market share. A higher value of *β* means more fierce competition between the two supply chain members. A higher $p_{r}^{SO}$ ($p_{m}^{SO}$) means a lower retail (direct sales) channel demand but a higher direct sales (retail) channel demand. A higher retail price of one supply chain member negatively affects its demand but positively affects the demand of the other supply chain member.

In the SL strategy, the manufacturer also sets up a direct online channel, but sells products to consumers through live streaming. The manufacturer’s live-streaming direct online retail price is $p_{m}^{SL}$, and the retailer’s traditional online retail price is $p_{r}^{SL}$. Different from the traditional online selling, the manufacturer needs to employ a streamer to sell the products in the live-streaming sales channel. Streamers with more followers may attract more potential consumers, but are also more expensive to employ [31]. In addition, increased promotion of live-streaming sales can also potentially boost demand. Both the choice of a streamer and the promotion of live streaming show the manufacturer’s live-streaming sales effort, represented by *e* [28]. The streamer’s sales skills *γ* also play a key role in live-streaming sales since the interaction between the streamer and consumers along with an effective presentation of the product can attract consumers to purchase [27]. Thus, the sales effort level and the sales skills both affect the market demand [32]. In the live-streaming sales channel, the market demand consists of two parts, one part being the original market demand affected by retail prices, and the other part being influenced by live-streaming sales depending on the live-streaming sales effort level and streamer’s sales skills. Following Zhang, Liu et al. and Du et al. [27,28], the demand functions of the retailer and the manufacturer are given by

$$D_{r}^{SL}=\theta a-p_{r}^{SL}+\beta p_{m}^{SL},$$ (3)

$$D_{m}^{SL}=(1-\theta )a-p_{m}^{SL}+\beta p_{r}^{SL}+\gamma e.$$ (4)

From Eq (4), a higher *γ* reflects better sales skills of the streamer. Furthermore, both higher live-streaming sales effort and higher streamer sales skills lead to higher sales in the live-streaming sales channel.

To avoid triviality, the unit production cost is assumed to be zero for the three strategies [33]. In the SL strategy, the live-streaming sales cost including hiring a streamer and promoting live-streaming sales can be assumed to be $\frac{\kappa }{2}e^{2}$, which is used widely in the existing literature [34], where *κ* is the live-streaming effort cost coefficient and a higher *κ* means the sales effort is more costly. The quadratic cost function is intended to reflect the fact that the cost increases but at a decreasing marginal speed as the sales effort increases. A rational manager always aims to be within reach, with the resulting subsequent improvements being more difficult to make.

## 4. Models and equilibrium results

For the manufacturer and the retailer, the profit maximization models for the NS, SO and SL strategies are proposed to derive the optimal decisions. Specially, the two sequences of events in the models for the SO and SL strategies are as follows. The first sequence of events is when the retailer decides the retail price after the manufacturer decides the direct online retail price. The other sequence of events is when the manufacturer and the retailer decide the retail prices simultaneously. Sensitivity analyses are conducted to examine the impacts of the key parameters on the optimal decisions and the optimal profits.

### 4.1. The NS strategy—no direct sales channels

**Fig 2:**
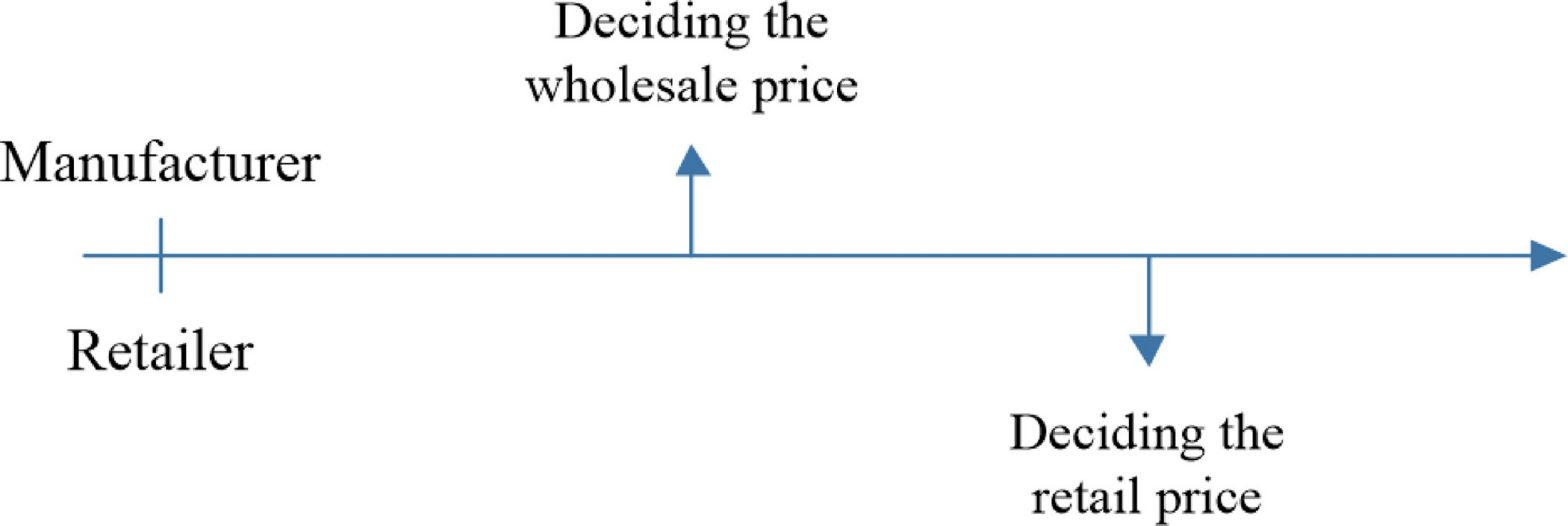
The sequence of events for the NS strategy.

The NS strategy as a benchmark is first considered when the manufacturer does not open a direct sales channel and only supplies products to the retailer. The optimal results of the benchmark are used to compare with those of the other two strategies. The manufacturer decides first the wholesale price *w*^*NS*^, and the retailer then decides the online retail price $p_{r}^{NS}$. The sequence of events for the NS strategy is depicted in Fig 2.

The profit maximization models of the manufacturer and the retailer can be described as

$$\underset{w^{NS}}{\max}\;\pi_{m}^{NS}=(a-p_{r}^{NS})w^{NS},$$ (5)

$$\underset{p_{r}^{NS}}{\max}\;\pi_{r}^{NS}=(a-p_{r}^{NS})(p_{r}^{NS}-w^{NS}).$$ (6)

The above optimization models (5) and (6) are solved using backward induction, and the optimal results for the NS strategy are then obtained. Thus, when the manufacturer does not open a direct sales channel, the optimal decisions for the manufacturer and the retailer are $w^{NS*}=\frac{1}{2}a$ and $p_{r}^{NS*}=\frac{3}{4}a$, respectively.

After substituting *w*^NS*^ and $p_{r}^{NS*}$ into Eqs (5) and (6), the optimal profits for the manufacturer and the retailer are derived as $\pi_{m}^{NS*}=\frac{a^{2}}{8}$ and $\pi_{r}^{NS*}=\frac{a^{2}}{16}$, respectively. These results show that the optimal pricing decisions and the optimal profits are affected only by the market potential, and the optimal prices and profits increase as the market potential increases.

### 4.2. The SO strategy—setting up a traditional direct online channel

**Fig 3:**
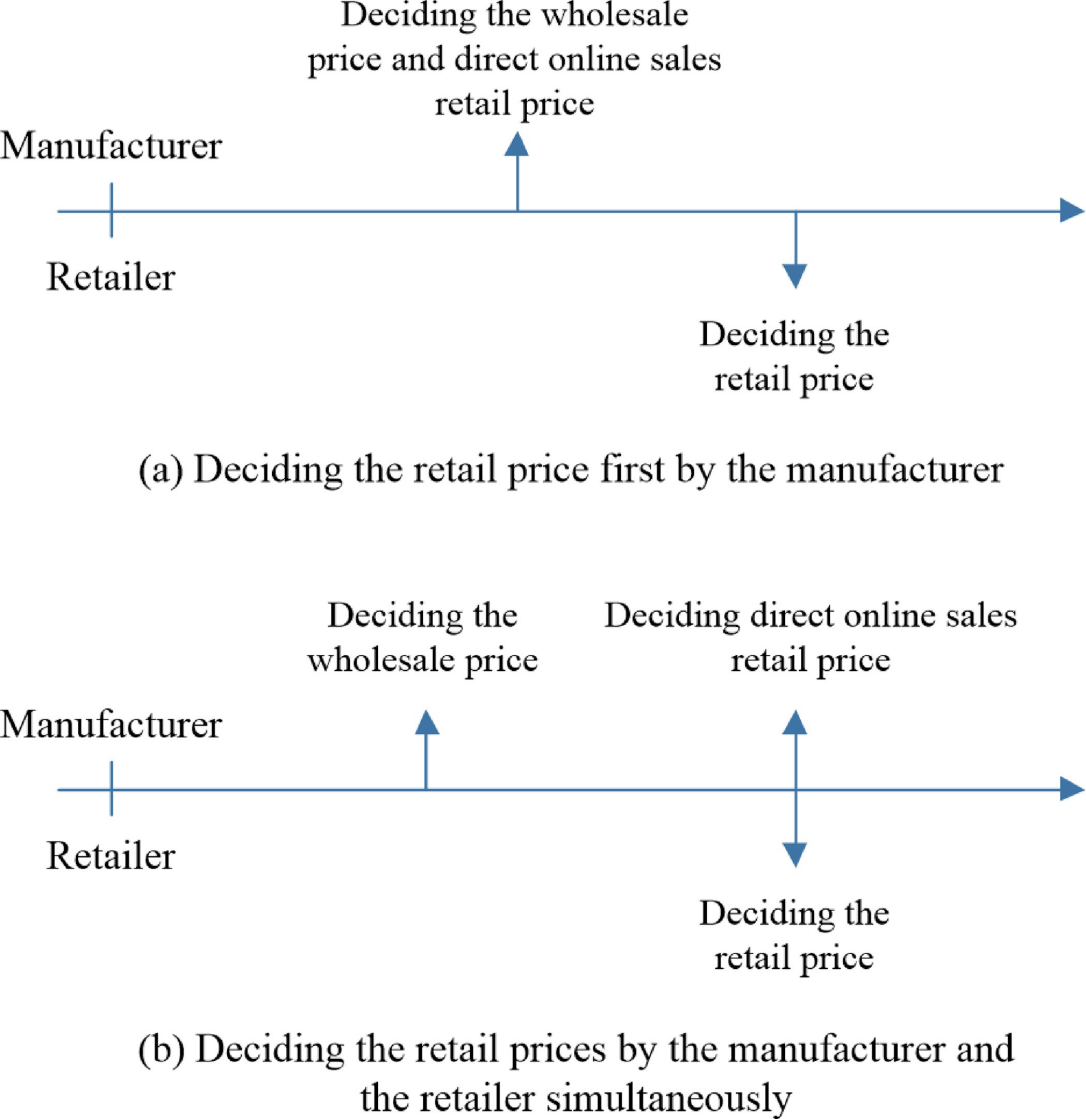
The sequences of events for the SO strategy.

For the SO strategy, the manufacturer not only supplies products to the retailer but also develops a traditional direct online sales channel to compete with the retailer. In practice, the following sequences of events may occur. (a) The manufacturer decides the wholesale price *w*^*SO*^ and the direct online retail price $p_{m}^{SO}$ first, and the retailer then decides the online retail price $p_{r}^{SO}$. (b) The manufacturer decides the wholesale price *w*^*SO*^ first and then works with the retailer to decide the retail prices $p_{r}^{SO}$ and $p_{m}^{SO}$ simultaneously. Thus, the above two sequences of events are considered for the SO strategy. The sequences of events for the SO strategy are depicted in Fig 3.

From Eqs (1) and (2), the profit maximization models of the manufacturer and the retailer can be described as

$$\underset{p_{m}^{Sa},w^{SO}}{\max}\;\pi_{m}^{SO}=p_{m}^{SO}D_{m}^{SO}+w^{SO}D_{r}^{SO},$$ (7)

$$\underset{p_{r}^{SO}}{\max}\;\pi_{r}^{SO}=(p_{r}^{SO}-w^{SO})D_{r}^{SO}.$$ (8)

As for the NS strategy, the above optimization models are solved through backward induction. The optimal solutions are then derived and stated in Proposition 1.

**Proposition 1.** When the manufacturer opens a traditional direct online sales channel, the optimal decisions are

- $p_{ma}^{SO*}=\frac{a(1-\theta +\beta \theta )}{2(1-\beta^{2})}$, $w_{a}^{SO*}=\frac{a[\theta +\beta (1-\theta )]}{2(1-\beta^{2})}$ and $p_{ra}^{SO*}=\frac{a[3\theta -\beta^{2}\theta +2\beta (1-\theta )]}{4(1-\beta^{2})}$ under the sequence of events (a);
- $p_{mb}^{SO*}=\frac{a[\delta (4-\beta^{2})+3\beta \tau +\theta \beta L_{1}]}{L_{1}(4-\beta^{2})}$, $w_{b}^{SO*}=\frac{a[\beta \delta +\tau ]}{L_{1}}$ and $p_{rb}^{SO*}=\frac{a[\tau (2+\beta^{2})+\beta \delta (4-\beta^{2})+2\theta L_{1}]}{L_{1}(4-\beta^{2})}$, with $\delta =(1-\theta )(8+\beta^{2})$, $\tau =\theta (8+\beta^{4})$ and $L_{1}=2(8+\beta^{2})(1-\beta^{2})$, under the sequence of events (b).

Based on Proposition 1, the optimal profits of the manufacturer and the retailer are $\pi_{ma}^{SO*}=\frac{a^{2}[2{\left(1-\theta +\beta \theta \right)}^{2}+\theta^{2}(1-\beta^{2})]}{8(1-\beta^{2})}$ and $\pi_{ra}^{SO*}=\frac{\theta^{2}a^{2}}{16}$, respectively, under the sequence of events (a), and are $\pi_{mb}^{SO*}=\frac{2a^{2}[\theta L_{1}-\tau (1-\beta^{2})](\beta \delta +\tau )}{L_{1}^{2}(4-\beta^{2})}+\frac{a^{2}[(4-\beta^{2})(\delta -\beta^{2}\delta -\beta \tau )+3\beta \tau +\theta \beta L_{1}][\delta (4-\beta^{2})+3\beta \tau +\theta \beta L_{1}]}{L_{1}^{2}{\left(4-\beta^{2}\right)}^{2}}$ and $\pi_{rb}^{SO*}=\frac{a^{2}\theta^{2}{\left(2+\beta^{2}\right)}^{2}}{{\left(8+\beta^{2}\right)}^{2}}$, respectively, under the sequence of events (b).

### 4.3. The SL strategy—setting up a live-streaming direct sales channel

**Fig 4:**
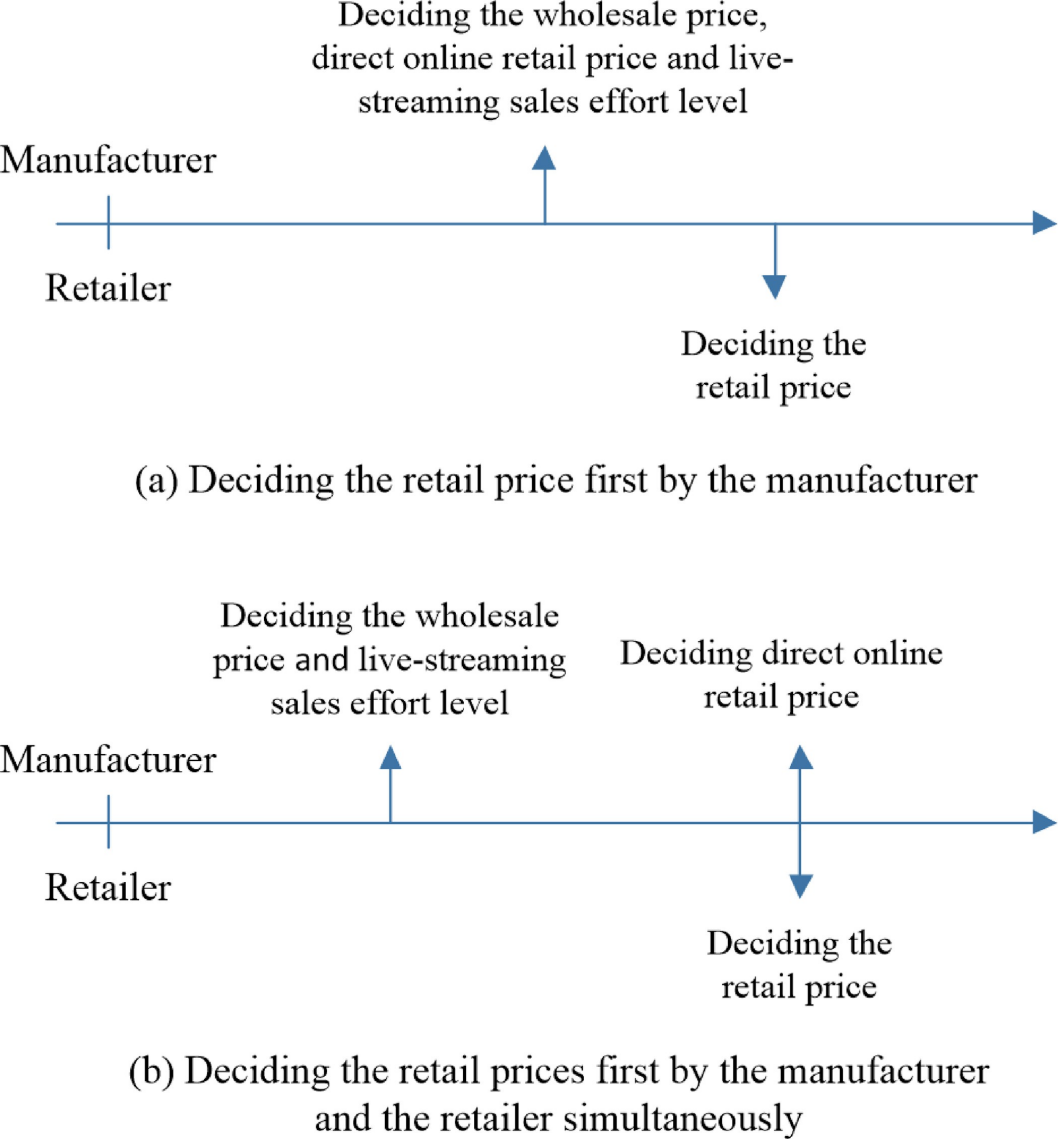
The sequences of events for the SL strategy.

For the SL strategy, the manufacturer sets up a direct channel but uses live streaming. The following sequences of events are considered. (a) The manufacturer decides the wholesale price *w*^*SL*^, the direct online retail price $p_{m}^{SL}$ and the live-streaming sales effort level *e* first, and the retailer then decides the traditional online retail price $p_{r}^{SL}$. (b) The manufacturer decides the wholesale price *w*^*SO*^ and the live-streaming sales effort level *e* first, and then works with the retailer to decide the retail prices $p_{r}^{SO}$ and $p_{m}^{SO}$ simultaneously. The sequences of events for the SL strategy are depicted in Fig 4.

From Eqs (3) and (4), the profit maximization models of the manufacturer and the retailer can be described as

$$\underset{p^{SL},w^{SL},e}{\max}\;\pi_{m}^{SL}=p_{m}^{SL}D_{m}^{SL}+w^{SL}D_{r}^{SL}-\frac{\kappa }{2}e^{2},$$ (9)

$$\underset{p_{r}^{SL}}{\max}\;\pi_{r}^{SL}=(p_{r}^{SL}-w^{SL})D_{r}^{SL}.$$ (10)

As for the NS and SO strategies, the above optimization models are solved through backward induction. The optimal solutions are then derived and stated in Proposition 2.

**Proposition 2.** When the manufacturer develops a live-streaming direct sales channel, the optimal decisions are

- $p_{ma}^{SL*}=\frac{a\kappa (1-\theta +\beta \theta )}{L_{2}},\;w_{a}^{SL*}=\frac{a[\left(2\kappa -\gamma^{2})\theta +2\beta \kappa (1-\theta \right)]}{2L_{2}},\;e_{a}^{*}=\frac{a\gamma (1-\theta +\beta \theta )}{L_{2}}$ and $p_{ra}^{SL*}=\frac{a[4\beta \kappa (1-\theta )+3\theta (2\kappa -\gamma^{2})-2\beta^{2}\kappa \theta ]}{4L_{2}}$ under the sequence of events (a);
- $p_{mb}^{SL*}=\frac{a[\kappa \delta +\kappa \beta \theta (10-\beta^{2})-\gamma^{2}\beta \theta ]}{L_{2}(8+\beta^{2})},\;w_{b}^{SL*}=\frac{a[\kappa (\beta \delta +\tau )-4\gamma^{2}\theta ]}{L_{2}(8+\beta^{2})},\;e_{b}^{*}=\frac{a\gamma (1-\theta +\beta \theta )}{L_{2}}$ and $p_{rb}^{SL*}=\frac{a[\kappa \beta \delta +\kappa \theta (12-2\beta^{2}-\beta^{4})-\gamma^{2}\theta (\beta^{2}+6)]}{L_{2}(8+\beta^{2})}$, with $L_{2}=2\kappa (1-\beta^{2})-\gamma^{2}$, under the sequence of events (b).

Based on Proposition 2, the optimal profits of the manufacturer and the retailer are $\pi_{ma}^{SL*}=\frac{a^{2}\kappa {\left(1-\theta +\beta \theta \right)}^{2}}{2L_{2}}+\frac{\theta^{2}a^{2}}{8}$ and $\pi_{ra}^{SL*}=\frac{\theta^{2}a^{2}}{16}$, respectively, under the sequence of events (a), and are $\pi_{mb}^{SL*}=\frac{a^{2}}{2L_{2}^{2}{\left(8+\beta^{2}\right)}^{2}}\{2\theta L_{2}(2+\beta^{2})[\kappa (\beta \delta +\tau )-4\gamma^{2}\theta ]+2\Phi [\kappa \delta +\kappa \beta \theta (10-\beta^{2})-\gamma^{2}\beta \theta ]-\gamma^{2}\kappa^{2}{\left(1-\theta +\beta \theta \right)}^{2}{\left(8+\beta^{2}\right)}^{2}\}$ and $\pi_{rb}^{SL*}=\frac{a^{2}\theta^{2}{\left(2+\beta^{2}\right)}^{2}}{{\left(8+\beta^{2}\right)}^{2}}$, respectively, with $\Phi =\kappa (1-\beta^{2})[\delta +\beta \theta (2+\beta^{2})]+3\gamma^{2}\beta \theta$, under the sequence of events (b).

## 5. Equilibrium analyses and comparisons

The impacts of the key parameters on the equilibrium solutions and profits are examined for the NS, SO and SL strategies. To investigate the optimal strategy for the manufacturer and the retailer, their optimal decisions and profits for the NS, SO and SL strategies are compared and analyzed. Numerical experiments are performed to verify the theoretical results. In the experiments, the parameter values are set to *a* = 10, *θ* = 0.5, *β* = 0.3, *γ* = 0.2 and *κ* = 0.3. These parameter values are used as defaults except for specifically mentioned otherwise.

### 5.1. Impact of important parameters

The important parameters investigated include the competition intensity *β*, the retailer’s market share *θ* and the streamer’s sales skills *γ*.

**Corollary 1.** Under the sequence of events (a), the impacts of the competition intensity *β* on the optimal results for the SO strategy are (1) $\frac{\partial p_{ma}^{SO*}}{\partial \beta }>0$; (2) $\frac{\partial w_{a}^{SO*}}{\partial \beta }>0$; (3) $\frac{\partial p_{ra}^{SO*}}{\partial \beta }\geq 0$ if $\theta \leq \frac{1+\beta^{2}}{{\left(1-\beta \right)}^{2}}$ and $\frac{\partial p_{ra}^{SO*}}{\partial \beta }<0$ otherwise; (4) $\frac{\partial \pi_{ma}^{SO*}}{\partial \beta }>0$; and (5) $\frac{\partial \pi_{ra}^{SO*}}{\partial \beta }=0$.

From Corollary 1, under the sequence of events (a), the direct online retail price, wholesale price and profit of the manufacturer all increase as the competition intensity *β* increases. The retail price of the retailer increases if the retailer’s market share falls below, and decreases if the retailer’s market share exceeds, a threshold $\frac{1+\beta^{2}}{{\left(1-\beta \right)}^{2}}$ as the competition intensity *β* increases. However, the retailer’s profit is not affected by the competition intensity. Intense competition causes the manufacturer to concern with consumer shifting from the direct sales channel to the retail channel. The manufacturer has a stronger incentive to raise the wholesale price to drive up the retail price since a high retail price may lead some consumers to shift from the retailer to the manufacturer. For instance, Apple exercises significant control over the pricing of its products, such as iPhone and iPad. It sets high wholesale prices to drive up retail prices and to maintain its brand premium image. This pricing policy can encourage consumers to buy directly from Apple stores or website rather than third-party retailers, which boosts its direct sales revenue (http://www.aeunike.lecture.ub.ac.id/files/2012/04/Case-Kel.13.pdf). Meanwhile, the manufacture raises the direct online retail price to ensure its profitability in fierce competition. Thus, this manufacturer pricing policy promotes an inward shift in the total demand, leading to an increased profit. Obviously, the manufacturer benefits from intense competition, and thus, should adopt strategies such as intensifying advertisement, improving product quality and providing personalized service to enhance competition with the retailer. Apple enhances its competitive edge with retailers through advertising strategies and brand promotion [35]. These strategies not only stimulate competition with the retailer but also sustain a competitive advantage in the market while meeting the evolving and diverse demands of consumers. Furthermore, the manufacturer should raise both the wholesale and retail prices to benefit from competition.

Moreover, when its market share is small, a high wholesale price causes the retailer to set an aggressive, i.e., high, retail price to avoid losses. In practice, the market share of a small emerging brand is small, e.g., when certain emerging technology product first enters the market. A high wholesale price causes retailers to set high retail prices to maintain a certain margin and to avoid sales losses, since this high wholesale price raises inventory risk. On the contrary, when its market share is large, the retailer has a stronger incentive to cut the retail price, causing the demand to shift from the manufacturer to the retailer. Walmart with a large market share may push for low retail prices while maintaining a small margin if suppliers set high wholesale prices. This pricing policy helps Walmart maintain low-price strategies to attract more consumers and to stay competitive. Under different market shares in tough competition, the retailer should use different pricing policies to dampen the competitor aggressiveness, and thus, maintain its profit. Therefore, with intense competition from the manufacturer, the retailer should reduce the retail price when its market share is sufficiently large.

**Corollary 2.** Under the sequence of events (a), the impacts of the competition intensity *β* on the optimal results for the SL strategy are (1) $\frac{\partial p_{ma}^{SL*}}{\partial \beta }>0$; (2) $\frac{\partial w_{a}^{SL*}}{\partial \beta }>0$; (3) $\frac{\partial e_{\alpha }^{*}}{\partial \beta }>0$; (4) $\frac{\partial p_{ra}^{SL*}}{\partial \beta }>0$; (5) $\frac{\partial \pi_{ma}^{SL*}}{\partial \beta }>0$; and (6) $\frac{\partial \pi_{ra}^{SL*}}{\partial \beta }=0$.

Corollary 2 shows that under the sequence of events (a), the live-streaming direct online retail price, wholesale price, live-streaming sales effort level, traditional online retail price and the manufacturer’s profit all increase as the competition intensity *β* increases. The retailer’s profit is not affected by the competition intensity *β*. When developing a live-streaming direct sales channel, a fierce competition induces the manufacturer to make more sales effort in the channel to attract more consumers, leading to higher costs. In real life, L’Oréal Paris invested heavily in pre-launch advertising through social media platforms, search engines, and short videos, e.g., Douyin (https://en.wikipedia.org/wiki/TikTok), the Chinese counterpart of TikTok, and Kuaishou (https://www.kuaishou.com/en), to attract viewers to live streaming. Manufacturers, such as Estée Lauder and Lancôme, hire the well-known streamer Li Jiaqi to sell their products through live streaming. To ensure products are prominently featured in Li Jiaqi’s live streaming sessions, the manufacturers typically pay high collaboration fees for the endorsement by the streamer. For example, a brand may pay additional fees to secure a “recommended spot” during the live streaming session, significantly increasing its marketing costs. Thus, the manufacturer has a strong incentive to raise the wholesale price, and to set an aggressive direct online retail price to increase profit. These strategies improve the total demand for the product, resulting in an increased profit. Thus, the manufacturer benefits from intense competition. The manufacturer is suggested to employ more influential streamers with high sales skills to promote live-streaming sales and to compete with the retailer. In 2024, Huawei teamed up with influential streamer Dong Yuhui to promote and sell smartphones through live streaming, that attracted a large number of viewers (https://wallstreetcn.com/articles/3710141). This strategy quickly boosted the sales and strengthened the position of Huawei in the smartphone market. Hence, the manufacturer should increase both wholesale and retail prices and should increase sales effort levels in a fierce competitive market to improve its profit.

The retailer, facing a high wholesale price, may pass some of the costs on to consumers, leading to an aggressive retail price, thereby maintaining its profit margins. The retailer adopts a different pricing policy in the SL strategy from that in the SO strategy when facing intense competition. This pricing policy may push the manufacturer to refrain from aggressiveness, protecting the retailer’s profit from the fierce competition. Thus, the retailer should always use a high-price strategy facing fierce competition when the manufacturer opens a live-streaming direct sales channel. Compared to the SO strategy, the retailer should shift its pricing policy in response to the manufacturer’s adoption of the live-streaming sales mode.

**Fig 5:**
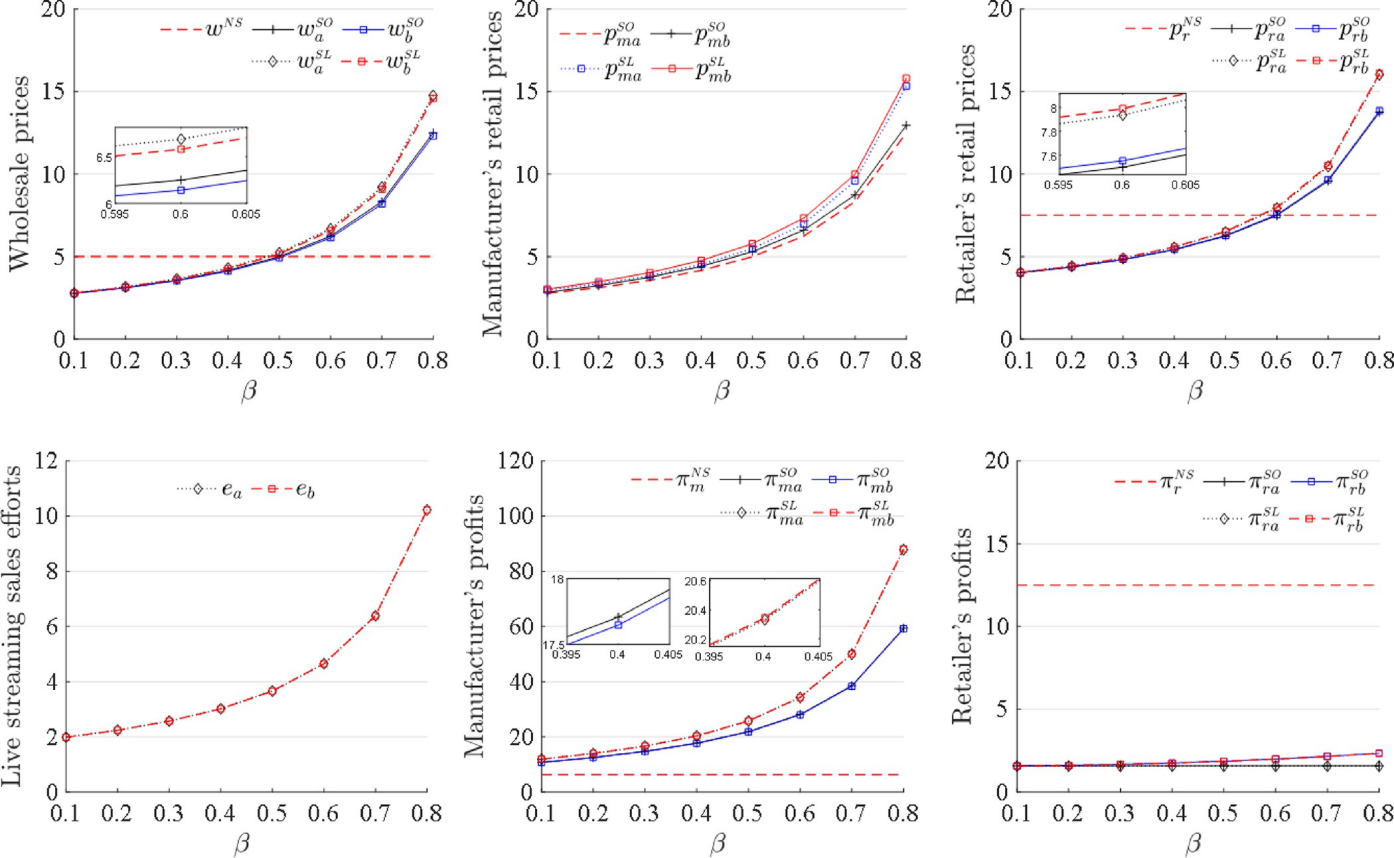
Changes in decisions and profits as *β* increases.

The results in Corollaries 1 and 2 are plotted in Fig 5. In addition, the relationships between the equilibrium decisions and the competition intensity *β*, and between the profits and the competition intensity *β*, under the sequence of events (b) are also plotted in Fig 5. The impacts of the competition intensity *β* on all equilibrium decisions and profits under the sequence of events (b) are similar to those under the sequence of events (a). These results show that the sequence of events does not affect the relationships between the equilibrium decisions and the competition intensity and between the profits and the competition intensity for the manufacturer and the retailer. The supply chain members are advised to use similar strategies under the sequence of events (b) to those under the sequence of events (a).

**Corollary 3.** Under the sequence of events (a), the impacts of the retailer’s market share on the optimal results for the SO strategy are (1) $\frac{\partial p_{ma}^{SO*}}{\partial \theta }<0$; (2) $\frac{\partial w_{a}^{SO*}}{\partial \theta }>0$; (3) $\frac{\partial p_{ra}^{SO*}}{\partial \theta }>0$; (4) $\frac{\partial \pi_{ma}^{SO*}}{\partial \theta }\geq 0$ if $\beta \leq \frac{3-2\theta }{\theta }$ and $\frac{\partial \pi_{ma}^{SO}*}{\partial \theta }<0$ otherwise; and (5) $\frac{\partial \pi_{ra}^{SO*}}{\partial \theta }>0$.

Corollary 3 shows that under the sequence of events (a), the direct online retail price decreases, but the wholesale price, the traditional online retail price and the retailer’s profit all increase, as the retailer’s market share increases. The impact of the retailer’s market share on the manufacturer’s profit is determined by the competition intensity. The manufacturer’s profit increases if the competition intensity falls below, but decreases if the competition intensity exceeds, a threshold $\frac{3-2\theta }{\theta }$ as the retailer’s market share increases. The introduction of the traditional online channel leads the manufacturer to seize a portion of the market share from the retailer, and thus, an increase in the retailer’s market share implies a decrease in the manufacturer’s market share. A larger retailer’s market share motivates the manufacturer to set a lower direct online retail price to seize market share, and to raise the wholesale price to compensate for the lower direct online retail price. This pricing policy does not always lead to increases in the total demand of the product. The manufacturer obtains more profit if competition mitigates, and less profit if competition intensifies. When the retailer has a large market share, the manufacturer using the traditional online sales mode benefits from mitigated competition, and thus, is advised to cooperate with the retailer to develop the market and promote the product, or to offer unique products and services in specific markets, effectively mitigating competition between them. For example, the partnership of Apple with Best Buy shows the benefit from a large retailer’s market share and network. Through their collaboration, Apple can leverage the market advantages of Best Buy to develop its market and to enhance its brand influence, and can also launch exclusive promotions and products at Best Buy to attract more consumers. This partnership effectively reduces competition between Apple and Best Buy and strengthens the market positions of both of them (https://www.retaildive.com/news/who-benefits-from-an-apple-best-buy-partnership/557610/). However, when competition is intense, the manufacturer using the traditional online sales mode benefits from its large market share, and thus, is advised to improve logistics efficiency in the direct online channel to ensure fast product delivery, meeting consumer demand for quick serve, and thereby expanding its market share. Dell’s ability to respond quickly to market demands by improving logistics efficiency enhances customer satisfaction and loyalty, expanding its market share (https://dfreight.org/blog/dell-supply-chain/).

The retailer with a large market share has great advantage when the manufacturer opens a traditional direct online sales channel, and may use this advantage to raise the retail price since consumers are often willing to pay more for trusted products. The retailer can use this pricing policy to increase its demand and improve its profit. Therefore, the retailer benefits from its market share, and is advised to use strategies similar to those of the manufacturer to expand its market share.

**Corollary 4.** Under the sequence of events (a), the impacts of the retailer’s market share on the optimal results for the SL strategy are (1) $\frac{\partial p_{ma}^{SL*}}{\partial \theta }<0$; (2) $\frac{\partial w_{a}^{SL*}}{\partial \theta }>0$; (3) $\frac{\partial e_{a}^{*}}{\partial \theta }<0$; (4) $\frac{\partial p_{ra}^{SL*}}{\partial \theta }\geq 0$ if $\beta \geq \frac{3\gamma^{2}}{2(3+\beta )(1-\beta )}$ and $\frac{\partial p_{ra}^{SL*}}{\partial \theta }<0$ otherwise; (5) $\frac{\partial \pi_{ma}^{SL*}}{\partial \theta }\geq 0$ if $\beta \geq {\tilde{\beta }}_{\pi }$ and $\frac{\partial \pi_{ma}^{SL*}}{\partial \theta }<0$ otherwise, with ${\tilde{\beta }}_{\pi }=\frac{-2\kappa (1-\theta )+\sqrt{\Delta_{1}}}{2\kappa \theta }$ and $\Delta_{1}=4\kappa [{\left(1-\theta \right)}^{2}+\kappa \theta (2-\theta )]+2\theta^{3}\gamma^{2}\kappa$; and (6) $\frac{\partial \pi_{ra}^{SL*}}{\partial \theta }>0$.

Corollary 4 shows that under the sequence of events (a), the live-streaming direct online retail price and the live-streaming sales effort level both decrease, but the wholesale price and the retailer’s profit increase, as the retailer’s market share *θ* increases. However, the relationships between the live-streaming direct online retail price and the retailer’s market share and between the manufacturer’s profit and the retailer’s market share depend on the competition intensity. The live-streaming direct online retail price and the manufacturer’s profit increase if the competition intensity exceeds, and decrease if the competition intensity falls below, a threshold as the retailer’s market share increases. When the manufacturer develops a live-streaming direct sales channel, a larger retailer’s market share causes the manufacturer to make less sales effort to mitigate losses. Despite the high sales effort level, the live-streaming direct channel may fail to attract more consumers due to its small market share. Moreover, a high sales effort level causes a high cost for the manufacturer. Hence, the manufacturer has a stronger incentive to cut the live-streaming direct retail price and raise the wholesale price to expand its market share. When the competition is intense, this pricing policy increases the product demand, dampens the competitor aggressiveness, and thus, improves the manufacturer’s profit. On the contrary, when the competition is mild, this pricing policy leads to a low manufacturer’s profit. When the retailer has a large market share, the manufacturer adopting a live-streaming direct sales channel benefits from intense competition and is advised to adopt this pricing policy early to stimulate competition with the retailer. However, when competition is mild, the manufacturer benefits from its large market share and is advised to adopt this pricing policy early to expand its market share and to mitigate competition with the retailer.

The retailer with a large market share typically possesses a strong bargaining power, allowing it to adjust retail price based on its competitiveness. In practice, Walmart is one of the largest retailers in the world, with a substantial market share and strong bargaining power (https://www.vaia.com/en-us/explanations/business-studies/business-case-studies/porters-five-forces-walmart/). In the highly competitive retail market, Walmart uses its market strength to adjust pricing policies. The retailer may increase its retail price to boost its profit when competition is intense because consumers may be willing to pay high prices for products they trust. Conversely, the retailer may cut its retail price to attract more consumers, which helps it in maintaining a leading position when competition is mild. This pricing policy weakens the competition from the manufacturer, and leads to an increased retailer’s profit. These results imply that the retailer can benefit from a large market share, and thus, is advised to use the strategies stated earlier to expand its market share.

**Fig 6:**
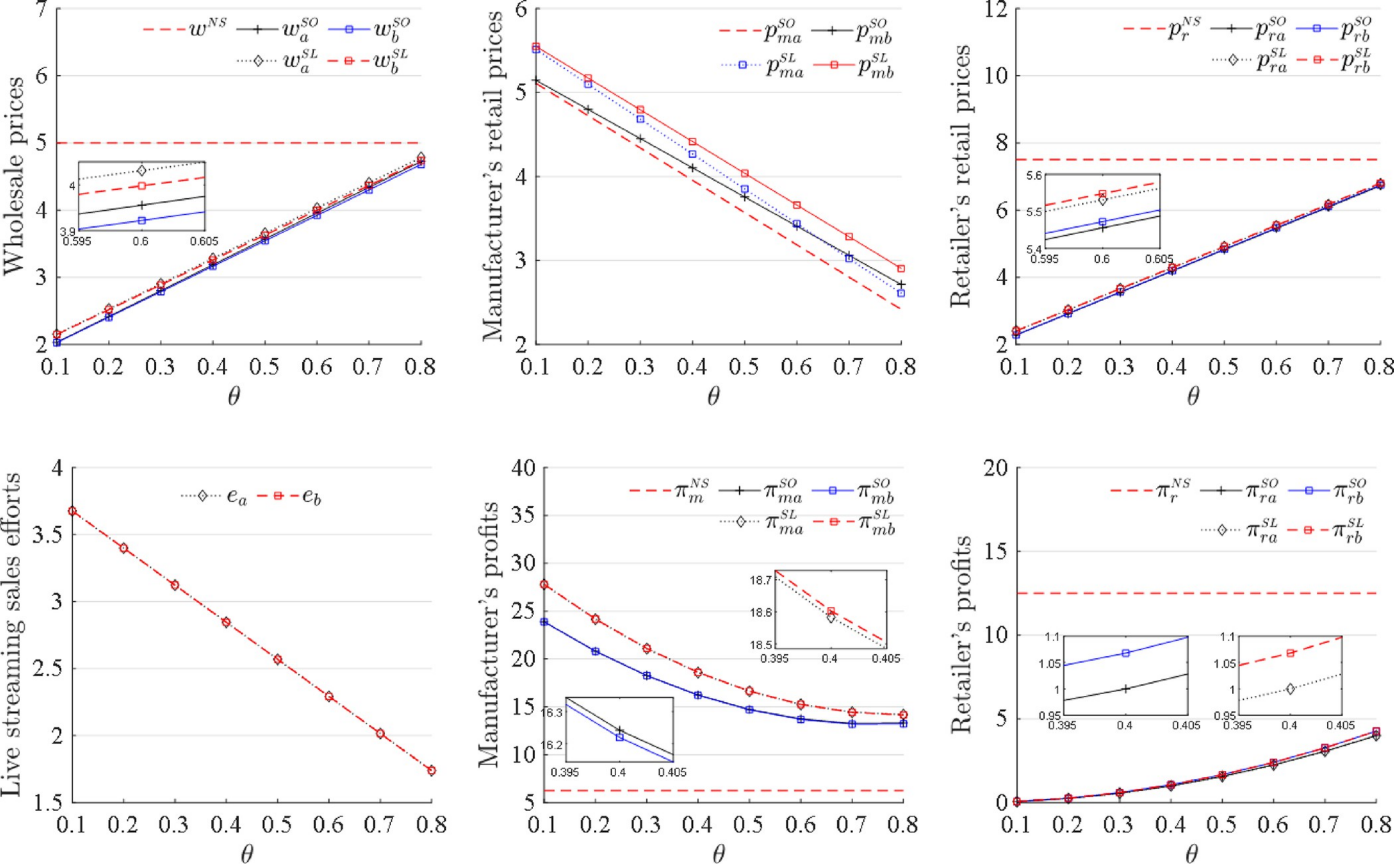
Changes in the decisions and profits as *θ* increases.

The results in Corollaries 3 and 4 are plotted in Fig 6. In addition, the relationships between equilibrium decisions and the retailer’s market share *θ* and between the profits and the retailer’s market share *θ* are plotted under the sequence of events (b). The variations in all equilibrium decisions and profits under the sequence of events (b) are similar to those under the sequence of events (a) as the retailer’s market share *θ* changes. Whether the manufacturer sets its direct online retail price first or cooperates with the retailer to set the retail prices simultaneously does not affect the optimal decisions and profits with changes in the retailer’s market share *θ*.

**Fig 7:**
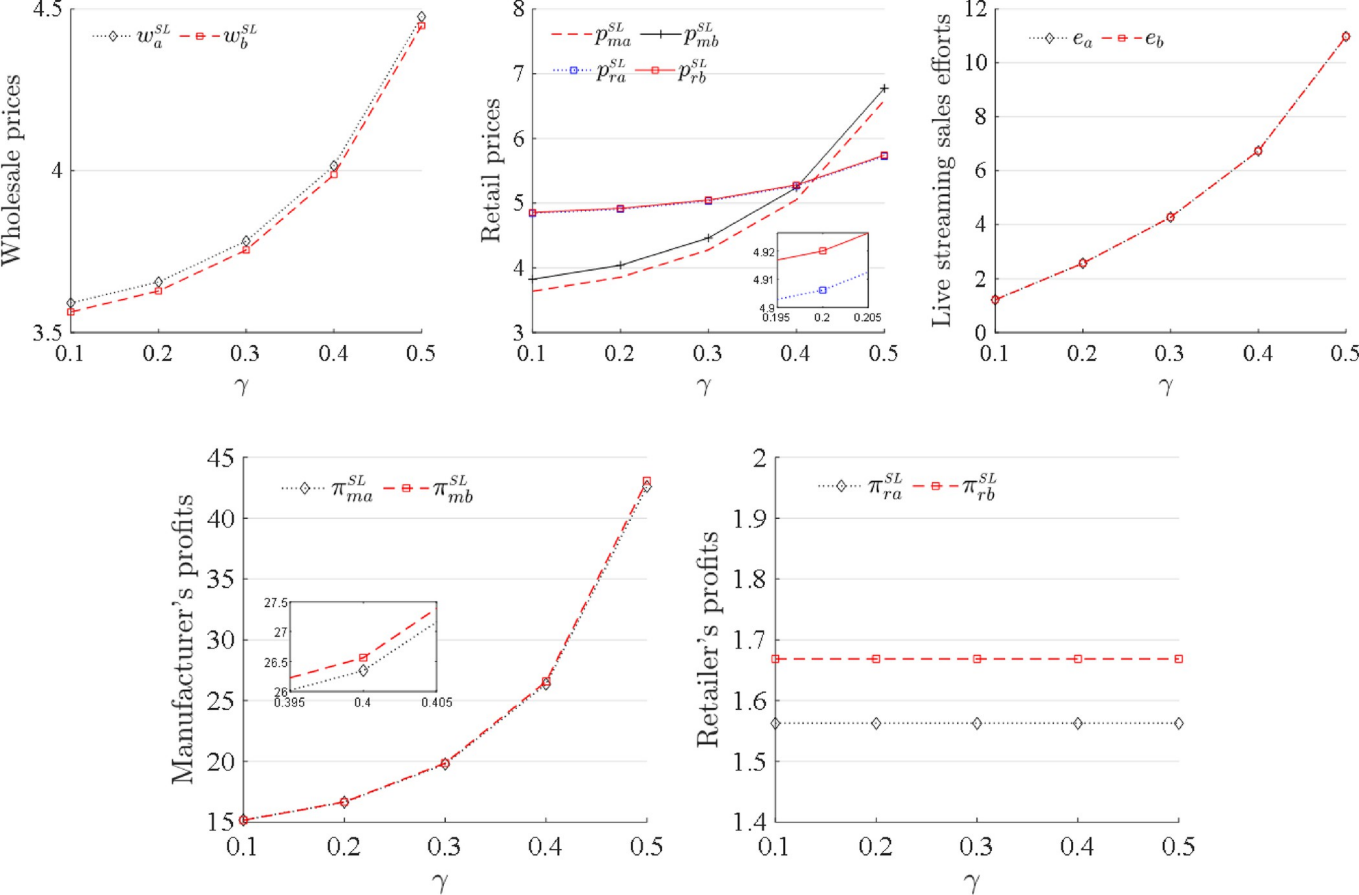
Changes in the decisions and profits as γ increases.

The impacts of the streamer’s sales skills *γ* on the wholesale prices, retail prices, sales effort levels and profits under the sequences of events (a) and (b) for the SL strategy are plotted in Fig 7. The wholesale and retail prices, live streaming sales effort levels and the manufacturer’s profits all increase as the streamer’s sales skills *γ* increase. The retailer’s profits are not affected by the streamer’s sales skills *γ*. The streamer with high sales skills *γ* can significantly enhance brand recognition and reputation. Through Li Jiaqi’s live streaming promotions, many brands have seen significant increases in visibility in a short period of time. For example, the beauty brand Florasis quickly gained substantial attention from consumers and boosted sales following Li Jiaqi’s endorsement through live streaming (https://www.digitaling.com/articles/981252.html). This increase in brand value, in turn, boosts market demand for the products. An increase in market demand drives up the wholesale and retail prices, as supply and demand dynamics take effect. In addition, as the streamer’s sales skills *γ* improve, sales volume increases significantly, promoting the manufacturer to recognize the vast potential of the live-streaming direct sales channel. To support and use the live-streaming sales mode, the manufacturer invests additional resources and makes additional effort, and thus, obtains additional profits. Obviously, the manufacturer benefits from the streamer with high sales skills *γ*, and is advised to employ streamers with high sales skills in the live-streaming sales channel. However, the retailer’s profit does not change with the increase in the streamer’s sales skills since the retailer adopts a high retail price policy to defend against the competition from the manufacturer. Hence, the retailer should increase its retail price when the manufacturer employs a streamer with high sales skills in the live-streaming sales channel.

### 5.2. Comparisons and analyses

To investigate the optimal dual-channel sales modes for the manufacturer and the retailer, the equilibrium decisions and profits for each supply chain member among the NS, SO and SL sales modes are compared and analyzed in this subsection. Furthermore, the equilibrium decisions and profits for the SO and SL sales modes between the sequences of events (a) and (b) are compared and discussed to investigate the suitable decision sequence for the supply chain members.

**Proposition 3.** The manufacturer’s optimal wholesale and retail prices for the NS, SO and SL sales modes satisfy

-
$p_{ma}^{SO*}\leq p_{ma}^{SL*}.$

- $w_{a}^{SO*}<w_{a}^{SL*}\leq w_{a}^{NS}$ if $\beta \leq {\hat{\beta }}_{w}$, $w_{a}^{SO*}\leq w_{a}^{NS*}<w_{a}^{SL*}$ if ${\hat{\beta }}_{w}<\beta \leq {\bar{\beta }}_{w}$, and $w_{a}^{NS*}\leq w_{a}^{SO*}<w_{a}^{SL*}$ otherwise, with ${\bar{\beta }}_{w}=\frac{-(1-\theta )+\sqrt{\left(1-\theta )(5-\theta \right)}}{2}$ and ${\hat{\beta }}_{w}=\frac{-\kappa (1-\theta )+\sqrt{\kappa^{2}(1-\theta )(5-\theta )-2\gamma^{2}}}{2\kappa }$.

Proposition 3 compares the manufacturer’s pricing decisions among the NS, SO and LS sales modes. Proposition 3 (1) shows that the manufacturer sets a higher direct retail price in the LS sales mode than in the SO sales mode. The manufacturer needs to pay commissions to the streamer, fees to the platform, and other marketing and promotional expenses in the LS sales mode. For instance, the beauty brand Florasis pays a percentage, e.g., 10%, of the sales amount as commissions to streamers, pays a fixed fee or sales commission to the live streaming platform, and also invests heavily in advertising and other marketing activities (https://business.sohu.com/a/674038104_121463337). Hence, the manufacturer sets a higher direct online retail price in the LS sales mode than in the SO sales mode. Proposition 3 (2) shows that the wholesale price is the highest in the NS sales mode and is the lowest in the SO sales mode if the competition intensity is sufficiently low; is the highest in the SL sales mode and is the lowest in the SO sales mode if the competition intensity is moderate; and is the highest in the SL sales mode and is the lowest in the NS sales mode otherwise. These results show that the wholesale price in the SL sales mode increases as the competition intensity increases. The competition is more intense in the SL sales mode than in the other two sales modes. However, the high costs and the fierce competition lead to an increase in investment in the live-streaming sales channel. Thus, the manufacturer sets the highest wholesale price to offset the high cost in the SL sales mode among the three sales modes.

**Proposition 4.** The optimal retail prices of the retailer satisfy $p_{ra}^{SO*}<p_{ra}^{SL*}\leq p_{ra}^{NS*}$ if $\beta \leq {\hat{\beta }}_{p},\;p_{ra}^{SO*}\leq p_{ra}^{NS*}<p_{ra}^{SL*}$ if ${\hat{\beta }}_{p}<\beta \leq {\bar{\beta }}_{p}$, and $p_{ra}^{NS*}\leq p_{ra}^{SO*}<p_{ra}^{SL*}$ otherwise, with ${\bar{\beta }}_{p}=\frac{-(1-\theta )+\sqrt{2(1-\theta )(5-2\theta )}}{3-\theta }$ and ${\hat{\beta }}_{p}=\frac{-2\kappa (1-\theta )+\sqrt{2\kappa (1-\theta )[4\kappa (5-2\theta )-3\gamma^{2}(3-\theta )]}}{2\kappa (3-\theta )}$.

Proposition 4 compares the retail prices of the retailer among the NS, SO and SL sales modes. These relationships in the retail prices are similar to those in the wholesale prices. As stated above, the manufacturer sets the highest wholesale price in the SL sales mode among the three sales modes as the competition level increases. The retailer also sets the highest retail price in the SL sales mode since the retailer incurs the highest cost in this sales mode. In addition, the manufacturer sets the highest direct retail price in the SL sales mode, and hence, the retailer often raises the retail price to align its retail price with the direct online retail price, upholding brand image and market positioning.

These results show that the retailer should set the highest retail price in the NS sales mode when the competition intensity is low, and should also set the highest retail price in the other sales modes when the competition intensity is moderate to sufficiently high. In addition, the retailer should set the lowest retail price in the SO sales mode when the competition intensity is low to moderate, and should do the same in the NS sales mode when the competition intensity is sufficiently high.

Fig 5 also illustrates the comparisons of the retail prices for the manufacturer and the retailer when the competition intensity changes. The manufacturer always sets lower retail prices than the retailer does as the competition intensity increases to adjust market positioning and competitive strategy in the market so as to gain a larger market share and enhance brand influence in intense competition. In addition, the comparisons in Fig 6. show that the retail prices for the manufacturer and the retailer depend on the retailer’s market share. The manufacturer sets a direct online retail price higher than the retailer does when it has a large market share, and lower than the retailer does when it has a small market share. In practice, Dell uses its strong market position to set higher prices for high-end products on its official website than in retail stores to maintain its brand value(https://www.dell.com/community/en/conversations/customer-care/charging-of-more-money-than-the-price-on-the-official-website-for-the-same-item-reg/647f8d3df4ccf8a8dedbc2ab). However, when entering a new or highly competitive market and having a small market share, Dell offers retail prices lower on its official website than in retail stores to quickly gain market share and attract consumers. Furthermore, the manufacturer sets a higher direct online retail price than the retailer does to protect the retailer interests and maintain their cooperative relationships when the retailer market share is small. This practice can avoid direct competition with the retailer, and preserve the overall health of the supply chain. Conversely, a larger retailer’s market share induces the manufacturer to set a lower direct online retail price than the retailer does to attract more consumers to expand its market share.

**Proposition 5.** The optimal profits under the NS, SO and SL sales modes satisfy

1. $\pi_{ma}^{SO*}<\pi_{ma}^{SL*}\leq \pi_{ma}^{NS*}$ if $\beta \leq {\hat{\beta }}_{\pi },\;\pi_{ma}^{SO*}\leq \pi_{ma}^{NS*}<\pi_{ma}^{SL*}$ if ${\hat{\beta }}_{\pi }<\beta \leq {\bar{\beta }}_{\pi }$, and $\pi_{ma}^{NS*}\leq \pi_{ma}^{SO*}<\pi_{ma}^{SL*}$ otherwise, with ${\bar{\beta }}_{\pi }=\frac{-2\theta (1-\theta )+\sqrt{\left(1-\theta^{2})(4\theta -\theta^{2}-1\right)}}{1+\theta^{2}}$ and ${\hat{\beta }}_{\pi }=\frac{-4\kappa \theta (1-\theta )+\sqrt{2\kappa (1-\theta^{2})[2\kappa (4\theta -\theta^{2}-1)-\gamma^{2}(1+\theta^{2})]}}{2\kappa (1+\theta^{2})}$, for the manufacturer;
2. $\pi_{ra}^{SO*}=\pi_{ra}^{SL*}<\pi_{ra}^{NS*}$ for the retailer.

Proposition 5 (1) compares the manufacturer’s profits among the NS, SO and SL sales modes. The manufacturer obtains the highest profit in the NS sales mode when the competition level is sufficiently low, and in the SL sales mode when the competition level is moderate to high. The manufacturer makes less sales effort in the SL sales mode, preventing the dual channel from generating additional profit when the competition level is sufficiently low, and thus, its profit is lower in the SL sales mode than in the NS sales mode. As the competition level increases, the manufacturer makes more sales effort in the SL sales mode, attracts more consumers, and thus, obtains the highest profit in the SL sales mode. Therefore, the manufacturer should use the strategies mentioned above to stimulate competition with the retailer when developing dual channels, and should choose the SL sales mode when competition is fierce.

Proposition 5 (2) shows that the retailer’s profit from the NS sales mode is always higher than those from the SO and SL sales modes. The manufacturer seizes the market share from the retailer when opening direct sales channels, and thus, the retailer’s profit decreases. The retailer’s profit remains the same whether the manufacturer introduces a traditional online or a live-streaming direct online sales channel. This result shows that the SO and SL sales modes do not affect the retailer’s profits because the retailer adopts different pricing policies to address the impacts of the manufacturer’s different direct sales channels. Therefore, the retailer should cooperate with the manufacturer when it does not open a dual channel. From Proposition 5 (1) and (2), when competition intensity is sufficiently low, both the manufacturer and the retailer obtain the highest profits when the manufacturer does not develop a dual channel, creating a win-win situation. Furthermore, Fig 5 demonstrates the results of Proposition 3 (2), Proposition 4 and Proposition 5 (1), and Figs 5 and 6 demonstrate the result of Proposition 5 (2). Fig 6 also illustrates the comparisons of the manufacturer’s profits among the NS, SO and SL sales modes as the retailer’s market share changes. The manufacturer obtains the highest profit in the SL sales mode and the lowest profit in the NS sales mode when the retailer has any market share. These results show that the retailer’s market share does not affect the relationship of the manufacturer’s profits among the different sales modes. The manufacturer attracts more consumers in the SL sales mode than in the SO sales mode regardless of the retailer’s market share. Thus, the manufacturer should always use the SL sales mode regardless of the retailer’s market share.

**Proposition 6.** The optimal decisions and profits with the sequences of events (a) and (b) satisfy

(1) $p_{ma}^{SO*}>p_{mb}^{SO*},\;w_{a}^{SO*}>w_{b}^{SO*},\;p_{ra}^{SO*}>p_{rb}^{SO*}$ and $\pi_{ra}^{SO*}<\pi_{rb}^{SO*}$ under the SO sales mode.
(2) $p_{ma}^{SL*}<p_{mb}^{SL*},\;w_{a}^{SL*}>w_{b}^{SL*},\;e_{a}^{*}=e_{b}^{*},\;p_{ra}^{SL*}>p_{rb}^{SL}$ and $\pi_{ra}^{SL*}<\pi_{rb}^{SL*}$ under the SL sales mode.

Proposition 6 (1) shows that the manufacturer sets higher direct online retail price and wholesale price, and the retailer sets higher retail price, under the sequence of events (a) than under the sequence of events (b) in the SO sales mode. The retailer’s profit is lower under the sequence of events (a) than under the sequence of events (b). The retailer benefits from jointly determining the retail prices with the manufacturer in the SO sales mode. By collaborating on pricing decisions with the manufacturer, the retailer can promptly adjust retail prices in response to market demand changes and competition dynamics. This flexibility enables the retailer to effectively manage costs, ultimately leading to an increase in profit. Therefore, the retailer should negotiate with the manufacturer to jointly decide retail prices.

Proposition 6 (2) shows that the manufacturer sets a higher wholesale price and a lower direct online retail price, and the retailer sets a higher retail price, under the sequence of events (a) than under the sequence of events (b), in the SL sales mode. The manufacturer’s live-streaming sales effort levels are the same under both the sequences of events (a) and (b). The retailer’s profit is lower under the sequence of events (a) than under the sequence of events (b). Hence, the retailer benefits from jointly determining the retail prices with the manufacturer, and thus, both the supply chain members are advised to follow similar decision sequence in the SO and SL sales modes.

Figs 5 and 6 demonstrate the result of Proposition 6 and also compare the manufacturer’s profits under the two different decision sequences. The manufacturer’s profit is higher under the sequence of events (a) than under the sequence of events (b) in the SO sales mode, but is higher under the sequence of events (b) than under the sequence of events (a) in the SL sales mode. By deciding on the direct online retail price ahead of the retailer, the manufacturer can take the first mover advantage in the SO sales mode, resulting in higher profit. However, selling through live streaming allows for real-time interaction and immediate market feedback, and the manufacturer and the retailer can adjust retail prices promptly based on the current market demands and consumer responses, inducing the manufacturer to swiftly capitalize on sales opportunities to maximize profit. Thus, the manufacturer is suggested to determine the direct online retail price before the retailer does in the SO sales mode and collaborate with the retailer to determine the retail prices simultaneously in the SL sales mode.

## 6. Managerial implications

The insightful managerial implications derived from the proposed profit maximization models and the obtained equilibrium solutions are provided in this section.

1. When the retailer has a large market share, the manufacturer should set a low direct online retail price and a high wholesale price in the SO and SL sales modes, and also should make more sales effort in the SL sales mode. Correspondingly, the retailer should set a high retail price when the manufacturer adopts the SO sales mode. However, they should shift from low-price to high-price policies if the competition intensity is sufficiently high when the manufacturer adopts the SL sales mode. When the competition is fierce, the manufacturer should raise its wholesale price and its direct online retail price, and should also make a sales effort similar to that when the retailer’s market share changes. The retailer should raise its retail price under a certain condition when the manufacturer adopts the SO sales mode, and should always raise its retail price when the manufacturer adopts the SL sales mode.
2. The manufacturer should establish a strategic partnership with the retailer to jointly develop markets, promote products, and offer unique products and services in specific markets to effectively reduce competition when adopting the SO sales mode. In real life, Procter & Gamble and Walmart have formed a long-term strategic partnership to collaboratively develop and promote various consumer products such as laundry detergent and shampoo, effectively reducing competitive pressures between them [36]. However, the manufacturer should employ streamers with widespread influence and/or good sales skills and make more sales efforts to push competition with the retailer when adopting the SL sales mode. For instance, Estée Lauder, Lancôme, and other well-known brands collaborate with the prominent streamer Li Jiaqi to promote and sell their products on live-streaming platforms, thereby increasing market share and effectively enhancing their competitive edges (https://www.cbo.cn/wap/article/view/id/51170).
3. The manufacturer should use the above mentioned strategies to appropriately increase competition with the retailer if it adopts the SO or the SL sales mode, and should adopt the SL sales mode when the competition is sufficiently fierce. In practice, Tesla extensively uses live-streaming sales in the Chinese market, successfully promoting its electric vehicles, demonstrating the effectiveness for manufacturers to utilize live-streaming sales to enhance market share in competitive environments (https://www.163.com/dy/article/FB25DISR0511X1K9.html). However, the retailer should always collaborate with the manufacturer when it adopts the NS sales mode. With a sufficiently low level of competition, the manufacturer adopting the NS sales mode can create a win-win situation for both supply chain members.
4. The manufacturer should determine the direct online retail price before the retailer does when it adopts the SO sales mode, and should work with the retailer to determine the retail prices simultaneously when it adopts the SL sales mode. Whether the manufacturer adopts the SO or the SL sales mode, the retailer should negotiate with the manufacturer to determine the retail prices simultaneously.

## 7. Conclusions

In a two-tier supply chain consisting of a manufacturer and a retailer, the channel selection and pricing decisions are examined, where the manufacturer distributes products to the retailer who sells them to consumers. Three, i.e., not developing dual channels (NS), introducing a traditional online direct sales channel (SO) and introducing a live-streaming direct sales channel (SL), sales modes are proposed for the manufacturer. In the SO and SL sales modes, the manufacturer competes with the retailer on the retail prices and decides the direct online retail price under two decision sequences, i.e., (a) the manufacturer decides the direct online retail price before the retailer does, and (b) the manufacturer collaborates with the retailer to decide the retail prices simultaneously. For the three sales modes, profit maximization models are proposed, and the optimal pricing decisions are derived through backward induction. Sensitivity analyses are performed to examine the impacts of the key parameters on the equilibrium pricing decisions and profits. The optimal decisions and profits are compared for the different decision sequences among the three sales modes to analyze the appropriate sales modes for the two supply chain members. Finally, several numerical experiments are performed to verify the derived theoretical results and to obtain more results.

Some important findings are summarized as follows:

1. The manufacturer’s wholesale price and live-streaming sales effort level increase but its direct online retail price decreases as the retailer’s market share increases. The retail price decreases if the competition intensity is low and increases otherwise with the increase in the retailer’s market share. A high competition intensity leads to high wholesale and direct online retail prices and a high live-streaming sales effort level. The retail price increases if the retailer’s market share is low and decreases otherwise if the manufacturer adopts the NS sales mode, and always increases if the manufacturer does not adopt the SL sales mode, with the increase in the competition intensity. Good streamer sales skills lead to increases in the wholesale and direct online retail prices and in the sales effort level in the SL sales mode.
2. If the retailer’s market share is large, the manufacturer can benefit from the SO sales mode with mild competition, and from the LS sales mode with fierce competition. However, the retailer can always benefit from a large market share when the manufacturer develops a dual channel. A high level of competition can always benefit the manufacturer, but does not affect the retailer, whether the manufacturer adopts the SO or the SL sales mode. Good streamer’s sales skills benefit the manufacturer but does not affect the retailer.
3. The manufacturer can benefit from the NS sales mode when the competition intensity is sufficiently low, and from the SL sales mode when the competition intensity is sufficiently high. The retailer can always benefit more from the NS sales mode than from the other sales modes. Both the manufacturer and the retailer can benefit from the NS sales mode if the competition intensity is sufficiently low.
4. The manufacturer can benefit by deciding the direct online retail price ahead of the retailer in the SO sales mode, and by deciding the retail prices simultaneously with the retailer in the SL sales mode. The retailer can benefit by deciding the retail price after the manufacturer does when the manufacturer adopts the SO or the SL sales mode.

One future research direction following this study is to incorporate the decision maker’s preference behavior into the live-streaming direct online channel decisions in a supply chain, specifically considering consumer reference effect and the supply chain member loss aversion. Another future research direction is to consider demand uncertainty into the Stackelberg game models and use a data-driven approach to examine the joint ordering and pricing decisions. Finally, one more future research direction is to incorporate product return processing into a direct online sales channel or the retail channel to examine pricing and ordering decisions.

## Supporting information

S1 Appendix The appendix provides all proofs of propositions and corollaries. (DOCX)
