# Supplementary material for: Traditional online and live-streaming dual-channel strategies and pricing policies
Source: PLoS One. 2025 Jan 8;20(1):e0311385. doi: 10.1371/journal.pone.0311385 (PMC11709306; doi:10.1371/journal.pone.0311385)
Supplement: S1 Appendix — (DOCX) [file pone.0311385.s001.docx]

# Appendix

## Proof of Proposition 1

The second partial derivative of in equation (6) with respect to is , and thus, is concave in . By the first-order condition of , the optimal retail price in is derived as . After substituting into in equation , the second partial derivative of with respect to is , and thus, is concave in . After setting , and are then derived.

## Proof of Proposition 2

**Proof under the sequence of events (a)**

The second partial derivative of in equation with respect to is , and thus, is concave in . By the first-order condition of , the optimal retail price in and is derived as . After substituting into in equation , the second partial derivatives of with respect to and are , and . Thus, the determinant of the Hessian matrix of is . is jointly concave in and . By the firs-order condition of , and are then derived. Thus, the optimal decisions , and are derived.

**Proof under the sequence of events (b)**

The second partial derivative of in equation with respect to is , and thus, is concave in . The second partial derivative of in equation with respect to is , and thus, is concave in . After setting and , and are then obtained. After substituting and into in equation , the second partial derivative of with respect to is , and thus, is concave in . By the first-order condition of , , and are obtained.

## Proof of Corollary 1

The first partial derivatives of , , and with respect to are given by , , and , respectively. In addition, because of , then if and otherwise.

## Proof of Corollary 2

The first partial derivatives of , , and with respect to are given by , , and , respectively. In addition, because of , then if and otherwise.

## Proof of Proposition 3

The proof is similar to that of Proposition 2.

## Proof of Corollary 3

The first partial derivatives of , , , , and with respect to are given by , , , , and , respectively.

## Proof of Corollary 4

The first partial derivatives of , , and with respect to are given by , , and , respectively. In addition, because of , then if and otherwise. From , is obtained. The two roots of are and , with , and thus, if and otherwise.

## Proof of Proposition 4

(1) Because of , then is obtained.

(2) Comparing with , holds. With , one root of is , and thus, if , and if . Moreover, comparing with , holds. With , one root of is , and thus, if , and if . Finally, holds. From and , if , and then if . In summary, if , if , and otherwise.

## Proof of Proposition 5

Comparing with , is obtained. With , one root of is , and thus, if , and if . Moreover, comparing with , holds. With , one root of is , where . Thus, if , and if . Finally, holds. From and , if , and then if . In summary, if , if , and otherwise.

## Proof of Proposition 6

(1) The proof is similar to that of Proposition 5.

(2) Because of and , then is obtained.

## Proof of Proposition 7

(1) Because of , , and , then , , and are obtained.

(2) Because of , , , and , then , , , and are obtained.
